# Biomimicry Training to Promote Employee Engagement in Sustainability

**DOI:** 10.3390/biomimetics7020071

**Published:** 2022-06-03

**Authors:** Sarah J. McInerney, Peter H. Niewiarowski

**Affiliations:** Program in Integrated Bioscience, Department of Biology, The University of Akron, Akron, OH 44325-3908, USA; phn@uakron.edu

**Keywords:** corporate sustainability, biomimicry, employee engagement, sustainable innovation, training psychology, environmental psychology

## Abstract

Employees play a critical role in the success of corporate sustainability initiatives, yet sustained employee engagement is a constant challenge. The psychology literature states that to intrinsically motivate employees to engage in sustainability, there must be opportunity for employees to engage in practices that are directly relevant to their job duties. Traditional ad hoc initiatives such as Earth Week events, recycling challenges and so on, are not sufficient to derive this type of intrinsic motivation. Therefore, the goal of this study was to examine the psychological impact of a biomimicry sustainable innovation training program, to intrinsically motivate R&D employees to reconnect with nature and identify whether this promotes creative thinking and employee engagement. Due to COVID-19 restrictions, the current study conducted virtual workshops with R&D employees and demonstrated that biomimicry training was intrinsically motivating to employees and was valued as a practice that could be incorporated into R&D job duties. In conclusion, this study provides an adaptable procedural template for biomimicry training with a corporate audience. The results demonstrate a strong business case for organizations to experiment with biomimicry by illustrating its potential to create positive change across several business units beyond sustainable innovation to include human resources and sustainable marketing.

## 1. Introduction

Organizations are under constant pressure to become more environmentally sustainable due to the insufficient supply of natural resources, pressure from various stakeholder groups, and increasing regulations requiring the public disclosure of corporate environmental performance. Organizations are also being held to heightened expectations by their employees [1,2]. The 120 million private sector employees in the U.S. have a critical role to play in reducing waste, increasing energy and water efficiency, innovating sustainable packaging solutions, and taking other actions to achieve corporate sustainability goals. However, only 22% of employees are engaged in corporate sustainability initiatives [1,3]. In 2017, the National Environmental Education Foundation (NEEF) published their fifth report of their Business and Environmental Program to support companies that are implementing employee education and engagement programing focused on the environment, sustainability, and corporate responsibility [1]. This report identified the main driver of employee engagement in sustainability to be training that an employee can incorporate into their job duties. This is supported by the training psychology literature that postulates that an individual’s intention or motivation to transfer training is increased if the training is considered to be credible, practical, and needed [4] in regard to their specific job duties. Therefore, it is not a surprise that traditional employee engagement opportunities, such as recycling challenges, clean-up events, donation drives, etc., opportunities that do not directly relate to job duties, have not engaged employees [1,2,3,4].

Moreover, the success of sustainability initiatives are often dependent upon employees’ voluntary pro-environmental behavior [5,6], requiring employees to be intrinsically motivated to adopt pro-environmental practices and behaviors. Intrinsic motivation is the desire to perform an action because of interest and satisfaction derived from the action itself, rather than external rewards or incentives. This type of ‘intrinsic motivation’ is often overlooked within corporate cultures that more commonly focus on extrinsic motivators such as bonuses, merit increases, promotions, etc. Although these types of extrinsic motivators have been shown to promote behavior change in the short term, the behavior is often not sustained over time [7,8,9,10].

Given the complexity of sustainability initiatives within a corporate environment and the critical need for employee engagement for success, it is essential that employees are intrinsically motivated by opportunities to engage in sustainability initiatives at work. Intrinsic motivation involves three essential factors: autonomy, mastery, and purpose [8,9]. Autonomy is directly linked to the self-determination theory that considers peoples’ psychological need for self-direction [11]. Autonomous workers who have more control over the work they do and how they do it have been shown to have higher job satisfaction and better job performance [8]. Mastery contributes to our inner drive to learn and to make progress with the work we do, requiring a balance of challenge and achievement to foster improvement and growth. Finally, in terms of purpose, employees who find purpose in their work and create a connection to a larger cause unlock the highest level of motivation and improved performance [9]. Therefore, given the need for sustained pro-environmental behavior and the importance of intrinsically motivated employees to voluntarily engage in such behaviors, employee engagement opportunities for sustainability also need to embody these three essential elements.

Creating pro-environmental behavior change is a significant challenge of our time and has led to a growing body of literature demonstrating human behavior as the dominant influence on our climate [12,13,14]. Psychologists, anthropologists, and ecologists have long maintained that human connection with the natural world is a large determinant of an individual’s worldview and behavior [2,15,16]. Through decades of industrial and technological advancements, humans have adopted an anthropocentric worldview, holding the human species at heightened importance over the natural world. The current consumer culture, especially in the West, has endorsed this disconnection from nature and has led to continued environmentally detrimental behavior from a personal to industrial level and from a local to global scale. Sustainability scientists are highlighting the urgent need for human populations to reconnect with the natural world through more than just a physical reconnection, but the active development of cognitive, emotional, and biophysical connections to create positive human–nature interactions [17]. Research in the environmental psychology and creativity literature has shown that a heightened connectedness to nature increases our capacity for innovation and is indicative of holistic cognitive styles and pro-environmental attitudes and beliefs, which often lead individuals to self-identify as environmentalist and to adapt a more eco-centric worldview, leading to pro-environmental behavior [18,19,20,21]. Connectedness to natural environments has also been shown to have restorative effects on directed attention [22,23], enabling more focused attention and higher-level cognitive function such as creative problem solving [24]. This focused creative problem solving coupled with a pro-environmental belief and eco-centric worldview could be the behavioral change required to tackle the current climate crisis.

Luckily, the current employee population is undergoing a generational shift, with over 75% of the workforce to be comprised of the millennial generation by 2025. The Deloitte Millennial Survey over the last decade has consistently identified that this incoming employee population is committed to positive social and environmental change and is willing to decline employment opportunities that conflict with these values [25]. Consequently, employee engagement practices in sustainability have evolved from ad hoc events to strategic programs aimed at providing advanced sustainability education, harnessing sustainability as a foundation for innovation and a way to build a competitive advantage and attract the incoming workforce [1]. Given this incoming values-based employee population, the traditional HR metrics of employee engagement such as ‘job satisfaction’ and ‘advocacy’ need to progress to integrate social and environmental metrics from sustainability departments such as time and dollars spent engaging in specific activities focused on actionable positive change [1]. Therefore, according to the above research, to improve employee engagement in sustainability and to enhance pro-environmental behavior within the workplace, there is a need for credible and practical professional development training that is intrinsically motivating to employees and enables them to reconnect to the natural world in a way that is directly relevant to their professional work.

One such opportunity is biomimicry, which is both a philosophy and interdisciplinary design approach that encourages us to learn from nature and to innovate through the emulation of biological forms, processes, patterns, and systems [26,27] to meet the challenges of sustainable development [28,29,30,31,32,33,34]. It is based on the premise that by understanding biological strategies, which achieve specific functions within a given context, and mimicking such in human technology, we can develop more environmentally sustainable solutions. Through biomimicry, participants from diverse fields interact with the natural world, whether that be through physical immersion, literature reviews, or scientific research, to discover the complexities of interconnected hierarchical systems to truly understand the strategies they wish to emulate. Thus, through a lens of innovation, biomimicry has the potential to reconnect corporate R&D practitioners to the natural world through content and a context that is directly relevant to their work, especially those whose job duties relate to new product development. Innovation driven by the challenges of sustainable development not only offers companies new ways to generate business value [35], but it also provides a direct link to employees’ values through the concept of ‘job purposing’, linking an organization’s purpose and an employee’s job to societal and environmental contribution [1,25], an approach that resonates heavily with the incoming eco-conscious generation. Biomimicry has shown its ability to drive environmentally sustainable solutions [36] and thus has demonstrated its credibility, practicality, and need given the current climate crisis and consumer demand for sustainable products. To add to that, biomimicry, compared to traditional sustainability engagement opportunities, also provides an opportunity to embed the three essential elements for intrinsic motivation: purpose, mastery, and autonomy. The focus of biomimicry to drive environmentally sustainable innovation through connecting innovation to the natural world provides a clear environmental purpose for practitioners. The interdisciplinarity of biomimicry requires practitioners to continuously learn new skills and ways of thinking, as the process fluidly transitions through design, biology, business, and engineering, therefore evoking our inner drive to learn, achieve, and grow. Finally, biomimicry requires practitioners to explore the natural world in search for biological strategies to inform design solutions; this requires independent research, giving practitioners autonomy over the process, to be guided by their own innate personal interest in nature. It is this type of independent exploration of the natural world that can lead to the active development of physical, cognitive, and emotional linkages required to reconnect people to the natural world [17,37] to promote pro-environmental attitudes and behaviors.

Biomimicry has shown its potential to drive sustainability-orientated innovation across a diversity of scales and disciplines [27,35,36,38,39]. Within a corporate setting, biomimicry is primarily siloed within R&D departments, valued as a design approach for sustainability-orientated innovation. Consequently, several areas of the biomimicry literature have supported this position by specifically focusing on the development of additional tools and strategies for innovation [40,41,42]. However, as previously discussed, the successful implementation of these tools, strategies, and ways of thinking is reliant upon employee’s behavior and their motivation to engage with such. Yet, the potential of biomimicry to drive environmentally sustainable behavior has not been explored to date. Therefore, the goal of this study was to examine the psychological impact of a biomimicry training program, to intrinsically motivate R&D employees to reconnect with nature and identify whether this promotes creative thinking and employee engagement. Demonstrating a positive impact would provide additional rationale to support the implementation of biomimicry within a corporate setting to not only drive sustainability-orientated innovation but to increase employee engagement. This study also adds to the small but expanding biomimicry training literature focusing on corporate audiences [36,40,43,44].

## 2. Materials and Methods

### 2.1. Experimental Procedure

R&D professionals from a consumer-packaged goods company were invited to participate in virtual biomimicry training. Participants were informed of the workshops at their monthly R&D meeting and encouraged to participate via email reminders. Participants were informed that the training would provide a general understanding of biomimicry, an interdisciplinary design approach, via a design challenge relevant to their current job duties. Continuous professional development training and workshops such as these are considered activities within the scope of regular job duties; thus, no extra compensation was provided. The company studied encompasses four R&D-specific business areas (SBAs); therefore, the design challenge portion of the training focused on a problem statement that was relevant to each SBA. The problem statements were selected through discussion with management of each SBA followed by further deliberation between the main author (S.J.M.) and a training facilitator, who is a full-time employee of the company, to ensure the problem statements were relevant and of similar difficultly for each SBA. As this study was conducted within a corporate setting, details of the problem statement, design challenge, and workshop outcomes are proprietary to the corporate entity, thus cannot be described due to confidentiality. The participants were informed that the training would consist of a 1 h tutorial followed by two 3 h workshops to be conducted virtually via Microsoft Teams over a one-week period with at least 1 day between sessions. This break between sessions provided time for participants to conduct individual research to explore biological strategies and their potential implementation into design solutions.

One SBA training session occurred per month from November to February (Table 1). Although the problem statements were relevant to each SBA, the training materials and process followed for the design challenge portion of the training were consistent across each SBA, as described in Section 2.3. Therefore, given that the experimental conditions relevant to the focus of this study, comparing the effects of a training intervention to no-training, remained consistent across SBAs, the results of each of the SBA training sessions were combined. All measures were administered via an online Qualtrics survey at three time intervals: pre-intervention (baseline measure), post-intervention (measure the effect of the intervention), and a retention survey 4 weeks from the post-intervention measure (measure the effect over time) (Table 1).

### 2.2. Participants

A total of 50 R&D employees, from across the four SBAs, collectively, volunteered to participate in the study; 30 employees completed the biomimicry training, referred to as the ‘training group’, and 20 employees formed the ‘no-training group’, who did not participate in the training. The no-training group completed the survey questions as they related to engagement in corporate sustainability initiatives available to R&D employees, without any expectation of participating in the biomimicry training. Corporate sustainability initiatives available to R&D employees included becoming a sustainability ambassador to organize events and initiatives, lunch and learn events to hear from experts in the field, and Earth Week activities such as product and financial donations, volunteer hours, electronic recycling, and paper shredding, etc. The organization has an established sustainability department; however, these were the opportunities available to employees working outside this department.

Traditionally, training class sizes within the current company range from 5 to 15 participants; thus, this was a representative sample size for this company. The table below displays the demographics of both groups (Table 2).

### 2.3. Biomimicry Training

All training sessions were developed and conducted by the lead author (S.J.M.) supported by an additional facilitator, a full-time employee at the company, to ensure a smooth presentation of the virtual content. The development of the biomimicry training was informed from a diversity of literature [26,40,43,44,45], open-access resources available through a variety of biomimicry channels such as The Biomimicry Institute, Biomimicry South Africa, Great Lakes Biomimicry, Ask Nature, etc., and participation in a variety of academic programs through the University of Akron Integrated Bioscience program and professional innovation training through the Biomimicry Research and Innovation Center. The training material developed for the current study followed a logical progression from least difficult to more complex information and was divided into three leading questions: (1) what is biomimicry, (2) what is the biomimicry design approach, and (3) how is it applied to a design challenge [26,36,40,41,43,45,46,47,48,49]. The biomimicry training was provided to the 30 employees within the training group, all of which attended both workshops 1 and 2. The training timeline and structure were as follows:

- A one-hour tutorial video, created by the lead author (S.J.M.), was provided the week prior to workshops.
  To access the video tutorial, participants had to complete the pre-survey as the reference link was provided on submission. This was used as a guideline to determine the level of engagement with the video tutorial and to ensure completion of the pre-intervention survey. The one-hour tutorial consisted of a 40 min introduction to the biomimicry design approach including several case studies; this portion was consistent across all SBAs. The final 20 min focused on the design challenge specific to each SBA problem statement, which followed the same structure: a 5 min introduction, 5 min exploring current hurdles and barriers to the R&D advancement of current solutions, followed by 10 min explaining the problem statement from a biomimicry perspective, outlining the problem statement and several functional representations of the problem. A functional problem statement was defined as beginning with an action verb, such as remove, transport, regulate, prevent, mix, supply, etc.; participants were made aware of the functional correspondents of the Engineering to Biology thesaurus [45] as a tool to support this process. Several potential functional representations of the problem were provided to aid participants’ exploration of biological models. Participants were encouraged to get outside, to explore local parks and biological institutions such as zoos, natural history museums, and botanical gardens, to watch documentaries, and explore several online tools such as Ask Nature, Google Scholar, YouTube, etc. Participants were tasked to specify a functionalization of the problem statement, to identify a biological model, and attempt to abstract design strategies to inform a design solution. A PowerPoint template to capture such information was provided to the training group.
- Workshop 1: A 3 h virtual workshop focused on Biological Model Exploration.
  The first two hours of the workshop consisted of biological model presentations and discussions. The lead author (S.J.M.) and the workshop facilitator presented for the first hour, describing several biological models in depth regarding potential abstracted design principles and solutions. In the second hour, the study participants shared the biological models and design strategies they had identified from the pre-work. Throughout these presentations, time was taken after each biological model to capture potential points of application to the problem statement.
  During the final hour, the abstracted design principles and potential points of application were further discussed to begin the transition into concept generation. The workshop ended with clear instruction for participants to review the material gathered in workshop one and to begin to brainstorm around concepts for the following workshop.
- Workshop 2: A 3 h virtual workshop focused on Concept generation.
  Materials from workshop one were revised, and a quick-fire round of brainstorming was completed to capture any initial build-on ideas participants had generated. The problem statement, as outlined by the SBA management, the specific hurdles, and spaces of opportunities identified were also revised. The next hour was focused on individual concept generation. Instructions to participants were to work on their own to generate concepts relevant to the design challenge statement. The lead author (S.J.M.) and the facilitator were available on the Teams chat if any assistance was required. For the final hour, participants presented their concept to the group followed by several minutes of discussion to capture additional build-on ideas and potential resources available that could support the advancement of the concepts.
  Finally, all participants were thanked for their participation and were directed to an online Qualtrics survey for the post-intervention survey.
- The post-survey was also distributed to the no-training group over the same timeframe. The no-training group did not participate in any workshop.
- The retention survey, also an online Qualtrics survey, was sent to both the training group and the no-training group 4 weeks after the post-survey was distributed.

### 2.4. Measures

All surveys across treatment groups and time points captured participants’ responses to the four measures, described below. The survey was administered at three time points, pre-intervention, post-intervention, and 4-week post-intervention. The pre-intervention survey contained 36 questions (Appendix A.1), 4 demographic questions, only asked in the pre-intervention survey, and 1 question to input a 4-digit participant code to enable comparisons across participants’ three survey time points and the following four measures. The four measures were two alternative use test questions [50], fourteen connectedness to nature questions [51], ten intrinsic motivation questions condensed from the Intrinsic Motivation Inventory [52,53], and five employee engagement questions derived from the 2017 National Environmental Education Foundation survey [1]. All questions were administered on a 5-point Likert scale (1 = strongly disagree, 5 = strongly agree).

The pre-survey questions were identical for both treatment groups focused on “*sustainable engagement opportunities*”. These acted as the baseline measure. The post-survey and 4-week survey for both groups repeated the same pre-survey questions, with demographic questions removed. The language used for the post-survey and the 4-week survey for the no-training group remained unchanged. The post-survey and 4-week survey for the training group contained the same survey questions as the no-training group with slightly adapted terminology to focus on the effect of the “biomimicry training”. This adaption was completed by simply adapting the terminology of the questions from a focus on ‘*sustainability engagement opportunities*’, which was presented to the no-training group, to focus on *‘biomimicry training’*, which was presented to the training group in their post-survey and 4-week retention survey (Appendix A.1).

#### 2.4.1. Intrinsic Motivation

Intrinsic motivation is the desire to perform an action because of interest, enjoyment, and satisfaction derived from the action itself, rather than external rewards. Intrinsic motivation is directly related to the self-determination theory (SDT) [11,52] which posits that those who are internally motivated following task participation are more likely to internalize the task and excel. Given the complexity of corporate sustainable initiatives and the importance of employee engagement for success, intrinsic motivation to engage in pro-environmental design and behavior both around a corporate campus but also in actual job duties such as sustainable product development is essential.

The Intrinsic Motivation Inventory (IMI) is an empirically validated, 45-item, multidimensional scale intended to measure the subjective experiences of participants following task participation [52,54]. The scale has six primary subscales that contain varied numbers of items within each, all of which have been shown to be factor analytically coherent and stable across a variety of tasks and conditions. Items within subscales overlap considerably and are often condensed to reduce redundancy. Shorter versions of these subscales have been used in past studies and have been shown to be reliable. The order effects of item presentation and the inclusion or exclusion of specific subscales have been shown to be negligible [52,53,54]. Therefore, it is common practice that experimenters choose subscales and specific items that are relevant to the issues they are exploring. The six subscales include Interest/Enjoyment, Perceived Competence, Effort, Value/Usefulness, Felt Pressure and Tension, and Perceived Choice. A seventh subscale labeled as Relatedness/Belonging was added in recent years but has not yet been validated. Like all self-report measures, caution is required when interpreting and reporting the findings, as the correlations between self-reports and actual behavior can be quite modest.

The present study was focused on the ‘usefulness and value’ of biomimicry training and the ‘interest and enjoyment’ in applying training to job duties to drive employee engagement in corporate sustainability initiatives. Therefore, these two subscales, interest and enjoyment, and value and usefulness, were used in this study. This led to the creation of a condensed ten-item IMI scale. The Interest/Enjoyment subscale is the primary measure of intrinsic motivation; thus, more items from this subscale are often used in adapted scales. The Interest/Enjoyment subscale (I/E) includes seven items regarding intrinsic motivation (i.e., “I enjoyed doing this assignment very much”), with two items reverse scored (i.e., “This assignment did not hold my attention at all”). Five items from this subscale were included in the condensed ten-item IMI survey, two of which were reverse scored.

The Value/Usefulness subscale (V/U) is used in internalization studies [11], with the intention to measure how participants internalize and become self-regulating with respect to activities that they experience as useful or valuable for themselves. This subscale includes seven items (i.e., “I believe doing this activity could be of some value to me), none of which are reverse scored. Five items from this subscale were included in the condensed ten-item IMI survey.

The ten-item survey was adapted for the training participants’ post- and 4-week retention surveys to measure the effect of the intervention. This adaptation was completed by replacing ‘*sustainability engagement opportunities*’ with ‘*biomimicry training*’, for example, I believe sustainability *engagement opportunities/biomimicry training* are of some value to me (Appendix A.1).

#### 2.4.2. Connectedness to Nature

The connectedness to nature scale (CNS) is a 14-item unidimensional scale developed by Mayer and Frantz (2004) to measure the trait level of one’s connectedness with nature [51]. Connection to nature is the way people identify with the environment around them and defines the relationships they form with the elements in those environments [55]. To begin to enhance pro-environmental behavior within the workplace, it is important to understand an employee’s connectedness to nature, as it is often correlated with a person’s level of environmental concern and their willingness to engage in pro-environmental behavior [56]. The CNS scale is widely accepted as reliable and valid by environmental psychologists [51,57,58] and since its publication in 2004 has been cited over 1500 times. Example items include “I often feel a sense of oneness with the natural world around me” and “I often feel disconnected from nature” (reverse scored). Participants rate how strongly they agree or disagree with each item on a 5-point Likert scale (1 = strongly disagree to 5 = strongly agree). The primary dependent variable is the degree to which respondent answers change from a pre- to post-test.

#### 2.4.3. Creative Thinking

The alternative use test (AUT) was designed by J.P Guildford in 1967 and is now a classic measure of divergent thinking [50]. The measure is dependent on the cognitive flexibility of the participant, to avoid fixation on one single category of use. Participants were given two minutes per item to list as many possible creative uses for two common household items per survey (e.g., “pen,” “towel”, “brick”, etc.). Participants were instructed to be creative, thereby encouraging responses of quality over quantity, thus making the scores valid indicators of individual difference as the participants were focused specifically on creative responses rather than providing a sheer quantity of responses [59,60]. Responses provided by the participants were rated by three independent raters (trained research assistants), using the subjective scoring method [59,61], an approach grounded in the consensual assessment technique of creativity assessment [62]. Three raters were trained to score responses for creative quality, using a 1 (not at all creative) to 5 (very creative) scale (Appendix A.2). This rating score was averaged across raters to yield a single rating for each participant. This rating is referred to as ‘creativity rating’ in subsequent analyses and discussion.

#### 2.4.4. Employee Engagement in Sustainability

The sustainability engagement index (SEI) was developed by the NEEF and was published in their 2017 report [1]. The SEI was created through discussions with corporate thought leaders, who included individuals from Baxter, Duke Energy, Genentech, Interface, PwC, SAP, and Spectrum Brands, regarding drivers for sustainability engagement and its relationship with employee engagement as traditionally defined by HR departments. The purpose of developing this index was to bridge the disconnection between HR and sustainability practitioners’ definition and means of measuring employee engagement. Sustainability practitioners describe “employee engagement” as the ability to motivate employees around sustainability goals and further their sustainability programs, measuring impact anecdotally or by the quantity of volunteer hours or dollars donated. HR professionals, on the other hand, describe employee engagement in terms of employees’ emotional commitment and discretionary effort, which is typically measured via an index approach using employees’ answers to survey questions such as whether employees would recommend the company as a great place to work or how proud they are to work at the company. The SEI is a novel yet crucial concept, as it enables the tracking of year over year performance and allows comparison among companies. The SEI is a 5-item measure rated on a 5-point Likert scale (1 = strongly disagree, 5 = strongly agree) and consists of the following five questions, which were adapted for the training participants’ post- and 4-week retention survey to measure the effect of the biomimicry training intervention. This adaptation was completed by replacing the terminology of ‘sustainability engagement opportunities’ with ‘biomimicry training’, captured in italics below.

1. *Sustainability engagement opportunities*/*biomimicry training* available at my company demonstrate practices that can be incorporated into my daily job activities.
2. Sustainability is critical to the future success of my company.
3. The *sustainability engagement opportunities*/*biomimicry training* available at work enhances my job satisfaction and overall feelings about the company.
4. In my opinion, my company’s *sustainability engagement opportunities*/*biomimicry training* enhances our brand in the community.
5. The *sustainability engagement opportunities*/*biomimicry training* available at my company demonstrate practices that can be incorporated into my personal life.

The SEI is not an empirically validated measure; therefore, statistical analysis was not completed given that firm conclusions cannot be drawn without prior validation, and the validation of the SEI was not the purpose of this study. Alternatively, the results are discussed qualitatively by comparing percent differences between the training and no-training group across the time scale for each individual item so as to be consistent with the NEEF 2017 report. Although there are several caveats to using this measure, it was deemed valuable to include in this study for several reasons: (1) it specifically addressed the overlap between HR and corporate sustainability that this study was targeting, (2) face and content validity was strong given the corporate setting, (3) the survey has been widely used by Fortune 500 companies representing over 100,000 employees [1], (4) it followed the same survey format as the several other instruments being implemented in this study, and (5) it was recommended by the company within which this study was conducted as an instrument they wished to implement.

## 3. Results

Mixed-model repeated measures ANOVAs (JMP 16 Pro) were used to test for treatment effects. A visual inspection of normal probability plots was used to verify assumptions of ANOVA. Treatment had two levels (training and no-training) and time had three levels (pre-, post-, and 4-week (post-intervention) surveys). A significant effect of time indicates differences among time points measured. All employees from both groups completed both the pre- and the post-survey measures (N = 20 for the no-training group, N = 30 for the training group), and 3 employees from each group did not complete the 4-week retention survey.

### 3.1. Intrinsic Motivation

**Figure 1:**
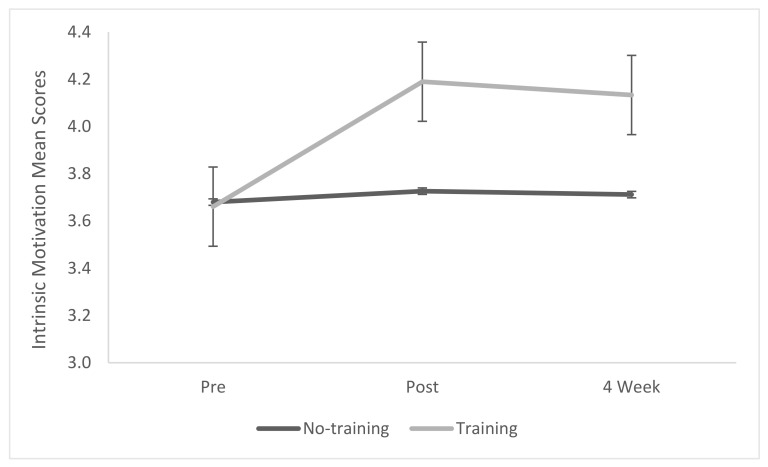
Intrinsic Motivation Inventory mean score.

The Intrinsic Motivation Inventory scale showed an internal reliability of α = 0.932 (Cronbach’s alpha = 0.932). A mixed-model repeated measures ANOVA of the whole measure, combining the subscales of interest and enjoyment and value and usefulness, revealed a marginally non-significant effect of treatment (*p* = 0.0552) with a significant effect of time (*p* = 0.0004, η^2^ = 0.163) and interaction (*p* = 0.0006, η^2^ = 0.152) (Table 3, Figure 1). Post hoc analysis via Tukey HSD Pairwise comparisons showed several indications that the biomimicry training group showed a significant increase in overall intrinsic motivation compared to the no-training group (*p* ≤0.0001), which was maintained over time (*p* ≤ 0.0001).

#### 3.1.1. Interest and Enjoyment (IE) Subscale for Intrinsic Motivation

**Figure 2:**
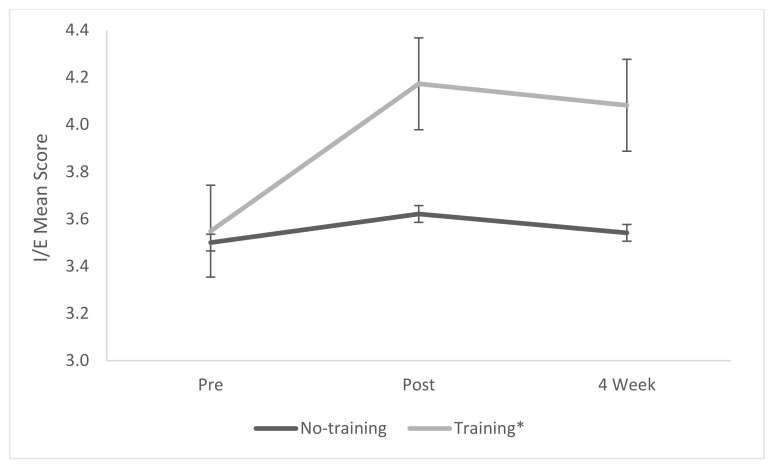
Mean scores for the Intrinsic Motivation Inventory subscale interest and enjoyment. * Statistically significant result.

A mixed-model repeated measures ANOVA showed a significant effect of treatment (*p* = 0.0267, η^2^ = 0.097), time (*p* = 0.0001, η^2^ = 0.183), and an interaction effect (*p* = 0.0023, η^2^ = 0.127) (Table 4 and Figure 2). Post hoc Tukey analysis showed that the group participating in the biomimicry training showed a significant increase in IE score compared to the control group (*p* ≤ 0.0001), which was maintained after 4 weeks (*p* ≤ 0.0001).

#### 3.1.2. Value and Usefulness (VU) Subscale for Intrinsic Motivation

**Figure 3:**
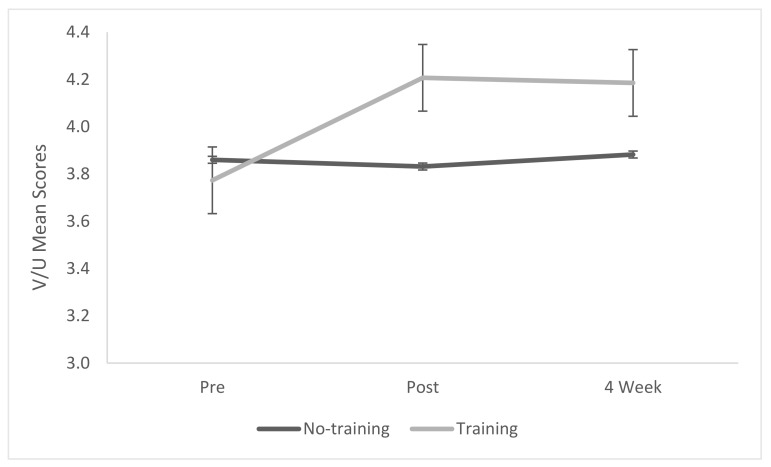
Mean scores of the Intrinsic Motivation Inventory subscale value and usefulness.

A mixed-model repeated measures ANOVA showed no main effect of treatment (*p* = 0.1465), but there was a significant effect of time (*p* = 0.0102, η^2^ = 0.098) and an interaction effect (*p* = 0.0017, η^2^ = 0.134) (Table 5 and Figure 3). Post hoc Tukey analysis showed that the biomimicry training group had a significant increase in value and usefulness (*p* ≤ 0.0001) compared to the no-training group, and this was sustained over time (*p* = 0.0004).

### 3.2. Connectedness to Nature

**Figure 4:**
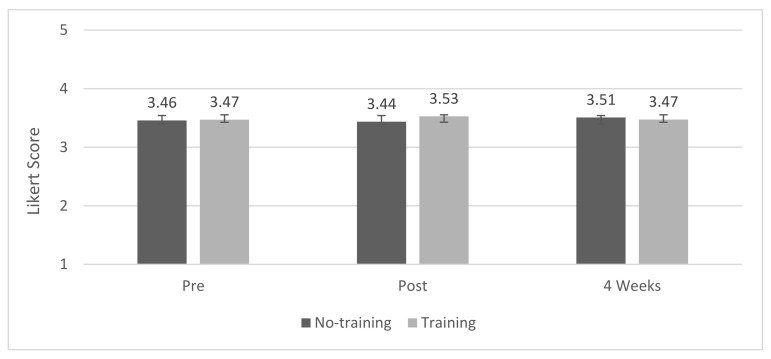
Connectedness to nature mean scores.

The connectedness to nature scale showed an internal reliability score of α = 0.875 (Cronbach’s alpha = 0.875). A mixed-model repeated measures ANOVA showed a non-significant effect of treatment (*p* = 0.8598), time (*p* = 0.5440), and the interaction between the two (*p* = 0.5253). All scores remained close to the midpoint of the scale with a mean score of approximately 3.5 at all measurement time points (Figure 4).

### 3.3. Alternative Use Test of Divergent Thinking

**Figure 5:**
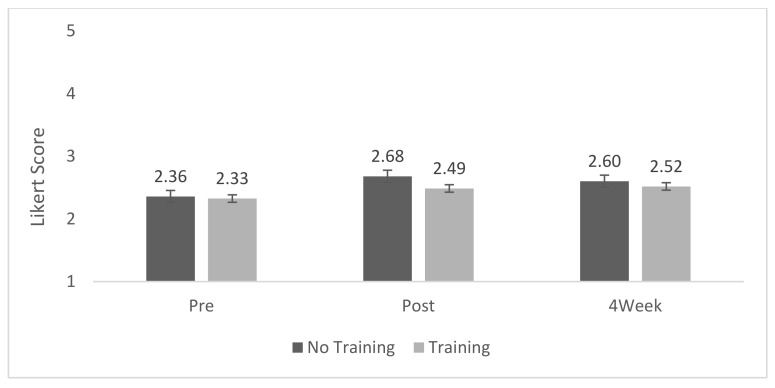
Alternative use test mean scores.

We estimated inter-rater reliability, which resulted in a coefficient alpha of 0.996. A mixed-model repeated measures ANOVA showed no main effect of treatment (*p* = 0.1800) or interaction (*p* = 0.3911), but there was a significant effect of time (*p* ≤ 0.0001). The alternative use test resulted in mean scores that were below or near the mid-point of the scale ranging from 2.3 to 2.7 across both groups and all measurement time points. Post hoc Tukey analysis showed that both groups showed a significant increase in divergent thinking score (*p* = 0.0031 and *p* ≤ 0.0001, biomimicry training group and no-training group, respectively), which was sustained over time (*p* = 0.0062 and *p* < 0.0091, η^2^ = 0.254, biomimicry training group and no-training group, respectively) (Table 6, Figure 5).

### 3.4. Sustainability Engagement Index

**Figure 6:**
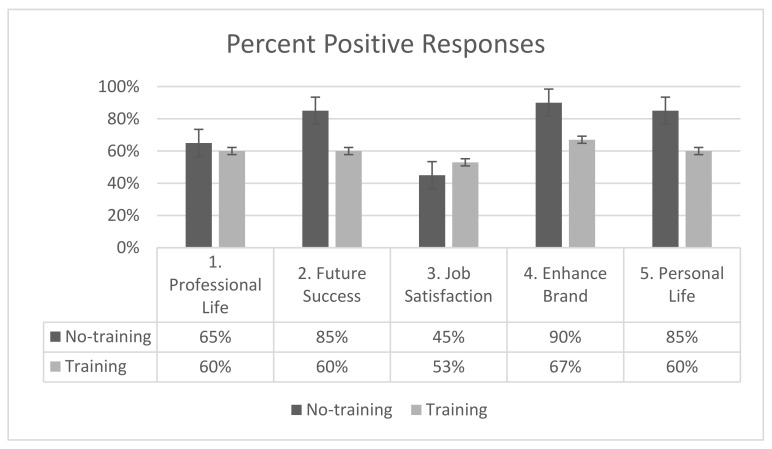
Sustainability engagement index percent positive scores at the pre-intervention stage.

**Figure 7:**
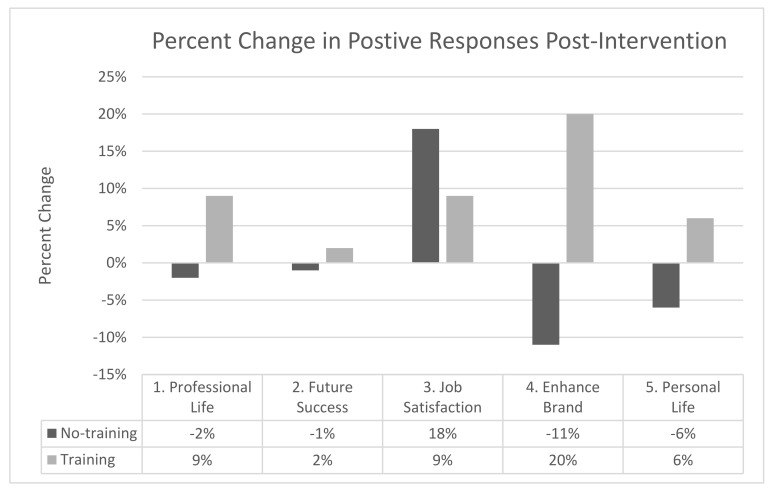
Percent change in positive responses for the sustainability engagement index items from the pre- to the post-intervention measurement time points.

**Figure 8:**
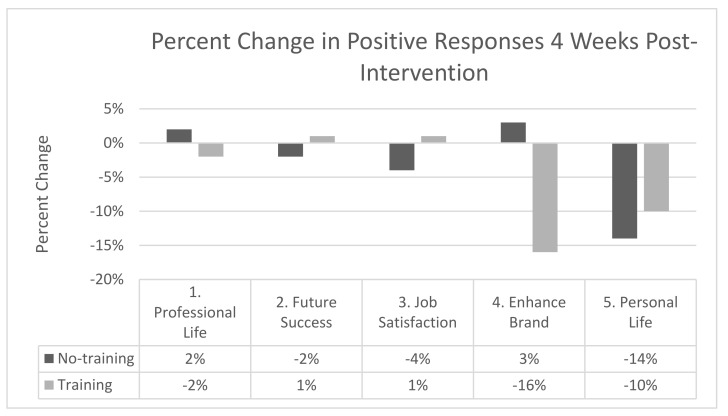
Percent change in positive responses for the sustainability engagement index items from the post- to the 4-week post-intervention measurement time points.

To allow for easier interpretation of the results across SEI items and to measure time points and maintain alignment with the NEEF 2017 report, the data were organized as follows. All positive responses (agree and strongly agree) were summed to give a representation of the study participants’ agreement with each survey question at the pre-intervention time point (Figure 6). The percent changes in positive responses for each question were calculated from the pre- to post-survey (Figure 7) and from the post- to the 4-week retention survey (Figure 8). The percent change of positive responses was calculated by subtracting the percent of positive responses of the later time point from the previous time point, and this was completed for each question and study group.

This pre-intervention time point analysis was completed to understand the level of sustainable engagement of study participants prior to the biomimicry training intervention. The biomimicry training was advertised to all participants as general training to understand the biomimicry design approach with an opportunity to apply learning to a problem statement relevant to their current job duties; it was not advertised as a sustainability professional development training opportunity. This was not an intentional feature of the study design. However, analysis at the pre-intervention stage provides insight into how R&D employees perceive the relationship between biomimicry and sustainability and the potential of biomimicry training to reach employees that might be less engaged in sustainability through innovation.

When comparing the two groups, the non-training group scored higher on the SEI than the training group. Compared to the training group, the no-training group more positively scored (selected agree or strongly agree) question 2 (future success of company, 85% vs. 60%), question 4 (enhance brand in community, 90% vs. 67%), and question 5 (transfer to personal life, 85% vs. 60%) (no-training vs. training, respectively). A similar percentage of participants from both groups positively scored question 1 (transfer to professional life, 65% vs. 60%, no-training vs. training), and a slightly higher percentage of the biomimicry training group positively scored question 3 (job satisfaction, 45% vs. 53%, no-training vs. training).

Overall, this demonstrated that employees are engaged in sustainability opportunities at work and either agree or strongly agree that such initiatives can be incorporated into their personal and professional lives (question 1 and 5, respectively, with 72% of study participants scoring these questions positively), is critical to the future success of the company (61% of participants scoring question 2 positively), and enhances the company brand in the community (77% of participants scoring question 4 positively). For question 3 regarding the ability of sustainability engagement opportunities at work to enhance job satisfaction and overall feelings about the company, 49% of study participants either agreed or strongly agreed with this statement.

At the post-intervention measurement time point, the percentage of participants that positively scored questions related to transfers into personal life (qt 1 and 5), the future success of company (qt 2), and enhancing the brand in the community (qt 4) increased for the biomimicry training group compared to the no-training group, which decreased, (question 1 (−2% vs. + 9%), 2 (−1% vs. + 2%), 4 (−11% vs. +20%), and 5 (−6% vs. +6%), (no-training vs. training group, respectively)). Question 3 in relation to job satisfaction increased for both the no-training group (+18%) and the biomimicry training group (+9%).

After 4 weeks, a retention survey was completed; positive responses for several questions remained stable from the post-measurement time point across both groups, e.g., questions related to transfers to professional life (qt 1, +2% vs. −2%), the future success of the company (qt 2, −2% vs. +1%), and job satisfaction (qt 3, −4% vs. +1%) (no-training vs. training, respectively). Question 4, regarding the ability to enhance the company brand in the community, was also relatively stable for the no-training group (+3%) but the biomimicry training group decreased in positive responses by 16%. Finally, for question 5, regarding the ability to incorporate practices learnt at work into one’s personal life, both groups decreased their positive responses: −14% vs. −10%, no-training vs. training group, respectively.

### 3.5. Correlation Table

Descriptive statistics, correlations, and reliabilities for the study variables are presented in Table 7, highlighting that several of these instruments are positively correlated although measuring a diversity of constructs. Sustainable engagement and intrinsic motivation were moderately positively correlated (r = 0.48), and both were weakly positively correlated with connectedness to nature (r = 0.32, r = 0.30, respectively). The alternative use test, a measure of cognitive creative ability, was very weakly negatively correlated with connectedness to nature and intrinsic motivation instrument and was very weakly positively correlated with sustainable engagement (r = −0.01, r = -0.01, r = 0.04, respectively).

## 4. Discussion

Overall, the current study demonstrated that biomimicry training can improve the intrinsic motivation of employees. This is critical, as intrinsic motivation is an essential pre-cursor to engagement and sustained behavior change [9,10], and the goal of training is to produce permanent cognitive and behavioral changes [63,64]. Therefore, in relation to sustainability programming, the goal is sustained pro-environmental behavior, a goal shared by many other domains given our current climate crisis. Sustained behavior change is dependent upon the motivation of an individual to engage in that behavior driven by interest and satisfaction derived from the action itself, rather than external rewards or incentives; this is intrinsic motivation [65]. The current study demonstrated that participants who received training in biomimicry had significantly improved intrinsic motivation compared to pre-training levels and significantly higher intrinsic motivation compared to those who did not participate in training across both subscales used, interest and enjoyment and value and usefulness. Biomimicry, innovation inspired by biological strategies, provided R&D participants with a unique opportunity to integrate their own interests and hobbies into their work in a relevant way through design and innovation. Hobbies such as watching nature documentaries, hiking, and visiting biological institutions such as zoos and natural history museums are several approaches that biomimicry practitioners engage in to explore biological systems for design solutions. The ability to engage in such a diversity of approaches provides autonomy to practitioners within the process, which is known to increase enjoyment and satisfaction in work [9,65], a traditional HR metric for employee engagement. This is supported by participants’ open comments stating that through the biomimicry training “I feel like I am incorporating biology back into my work life, which I appreciate”, with another stating that “it is fulfilling to see how we can incorporate what we are doing into part of the broader picture”, and other participants stating, “it does make me feel more satisfied to known I am impacting the environment in a positive way”.

Practitioners also valued the biomimicry training for its ability to promote design innovation, environmental sustainability, and systems thinking. This was stated in participants’ open comments sharing that the biomimicry approach “is another tool to think differently about problems and promote sustainability” and that “understanding the science behind nature is critical to unlocking applications to the problems to be solved”. Another participant added that the biomimicry training “helps to get in the habit of thinking outside the box and getting ideas from unlikely places”, while another stated “biomimicry provides close loop thinking” and “we can utilize this to decrease our carbon footprint”. This is a critical result, as it demonstrates the credibility and practicality of biomimicry, which are essential motivators to transfer training into actual job duties [4]. It also shows how biomimicry training can be directly relevant to employees across a diversity of business areas, innovation, R&D, sustainability, HR, and potentially others.

The potential of biomimicry to promote employee engagement in sustainability was supported qualitatively by the percentage of participants’ positive responses for all items within the SEI increasing for the biomimicry training group, while all but one item either decreased or remained stable for the no-training group (Figure 7) [1,3]. The one item that did increase for the no-training group was in relation to job satisfaction, which increased by 18% compared to the training group, which increased by 9%. This would suggest employees’ general satisfaction with their job and overall feelings about the company. However, the doubled increase for the no-training group could suggest that the training groups’ exposure to the biomimicry training sparked a realization regarding the lack of opportunities for employees to engage in sustainability in a way that is relevant to their job function. Hence, both groups scored their job satisfaction positively; however, the larger increase in positive responses came from the no-training group. Further research is required to substantiate this claim. The SEI item of specific importance was the relevance of the biomimicry training to be incorporated into an employee’s professional and personal life, which is recognized as the most effective way to increase employee engagement in sustainability [3,66,67]. Biomimicry was also in alignment with traditional sustainability engagement opportunities in its ability to enhance job satisfaction and overall feelings about the company, with over 60% of participants in both groups having a positive response to this statement at the post-measurement time point. The ability of biomimicry training to enhance company brands in the community showed the most substantial increase in positive responses at the post-measurement time point, increasing by 20% for the biomimicry training group and decreasing by 11% for the no-training group. This was an unanticipated positive result for the biomimicry training, which brought attention to the potential communicative and marketing value of biomimicry, especially within a corporate setting. Consumers’ nature-based values have been identified as having the most positive influence on consumers’ support for sustainable businesses and products [68]; therefore, biomimicry practice and product development could be a promising sustainable marketing strategy to enhance the company brand in the community. The ability of biomimicry to enhance several business units beyond sustainable innovation to include marketing and HR is vital, as it provides justification for a corporate audience to implement biomimicry as a credible job-specific employee training and engagement program focused on overall corporate sustainability goals rather than just sustainable product development.

Intrinsic motivation has also been positively correlated with creative performance [69], while exposure to natural environments has been demonstrated to promote creative cognitive styles [22,24]. This unfortunately was not supported in the current study. The divergent thinking measure, the alternative use test, remained at the mid-point of the scale for both groups across all three-time points. The same was observed for study participants’ connectedness to nature scores, which were just above average yet remained stable for both groups across all three time points. Given the virtual nature of the study, due to COVID-19 restrictions, the lack of effect on participants’ creative performance and connectedness to nature result was not surprising, yet it does pull into question the relevance of current corporate biomimicry training practices, which this study reflected to a degree. Currently, corporate biomimicry implementation and training is primarily focused on solving a specific R&D design challenge or problem statement. Perhaps the exploration of biological systems primarily for ideation or solution stimulus does not create a deep understanding of fundamental biological principles; rather, it only creates a surface-level connection between practitioners and the natural world. Constricting the biomimicry approach in this way may not be the most effective format to reconnect practitioners with the natural world to promote sustained pro-environmental behavior [18,19,20,21]. Research has shown that information provision is associated with action, meaning that individuals who are more knowledgeable about environmental issues are more connected to nature and show greater intention to engage in pro-environmental action [13]. Therefore, providing this fundamental biology education prior to biomimicry practice has the potential to foster a deeper understanding of, respect for, and therefore connection with the natural world. This could be accomplished in several ways that would not only be beneficial to biomimicry practitioners but also to the innovation process itself. Initial training can be given in fundamental biological principles either generally, from fields such as evolutionary biology, ecology, biodiversity, etc., or principles more relevant to the design challenge of interest, perhaps including biomechanics, biomaterials, thermal systems, biochemistry, etc. By acquiring fundamental biological knowledge, the learning curve to understand the R&D challenge from a biological perspective, the potential of certain biological solutions, and the environmental viability of design concepts could be drastically reduced. Overall, this would create a deeper understanding and appreciation for the natural world while expediting the design process. Practitioners could also collaborate with biological institutions from universities to natural history museum, zoos, botanical gardens, etc. Working in partnership with expert biologists and curators would increase the accuracy of the biological model identified and the abstraction of design strategies, vital steps for successful biomimicry innovation. Such collaborative partnership also provides the opportunity for participants to be immersed in natural settings with access to biological models and artifacts; this physical and cognitive immersion in a natural setting could be the missing link to create positive human–nature interactions while increasing employee engagement. Although this approach would require additional resources, the potential for sustainable innovation would be increased by connecting R&D practitioners to biological experts and physical models.

## 5. Limitations and Future Research

There were several limitations to the original intention of this study given the COVID-19 pandemic. The original intention for this study was to engage participants in immersive experiences exploring natural systems to identify potential biological solutions. This was adapted to be completed virtually, which added complexity to both the training development, delivery, and implementation process. Although past research had shown that virtual exposure to natural stimulus can increase connectedness to nature [58,70], this was unfortunately not demonstrated in this study. While participants within the training group were encouraged to get outside and physically explore natural systems, due to the virtual nature of the study and closures of biological institutions such as zoos, natural history museums, botanical gardens, etc., the degree of natural exposure could not be managed or controlled. General weather constraints may have also been a limiting factor, as the study was postponed from a summer to a winter completion date to provide time to adapt training materials to a virtual platform.

Due to the virtual nature of the biomimicry training and employee stress and uncertainty with the new work-from-home scenario, the decision was made to condense the training to a video tutorial and two 3 h sessions. This may have been too restricting and detrimental to the study, given that the study population was primarily biomimicry novices. Although training participants were encouraged to reach out to the lead author (S.J.M.) with any questions or for further discussion, this option was not implemented by any study participant. Therefore, training participants not only had limited physical immersion within a natural setting, but they also had no physical in-person contact, were not guided through the training material, and were not provided the opportunity to discuss key concepts with peers, unless on personal time. Thus, training participants were met with a steep learning curve to understand the biomimicry design approach itself, followed by the exploration of biological literature and materials. The brevity of the innovation sessions, 3 h to explore biological models and 3 h to develop concepts, may have also been too restrictive given the complexity of group work, brainstorming, and ideation in a virtual setting.

Beyond technological complexities, the lead author (S.J.M.) assumed that these brief sessions to ideate on a specific R&D problem statement did not provide adequate time or content for training participants to explore natural systems in enough depth to affect their connectedness to nature. Additionally, corporate audiences are often under-represented within academic research due to limited access and confidentially constraints, with most sample populations consisting of students or volunteers. Therefore, the lead author (S.J.M.) was concerned that the connectedness to nature scale, given its foundation in psychology, did not directly correlate with corporate language regarding environmental responsibility and stewardship. Given the static nature of the scale items, the terminology was not altered in the current study; therefore, future research implementing an instrument with a corporate-orientated vernacular may be better received by a corporate audience. Additionally, this study demonstrated that the sustainable engagement index is a valuable tool producing informative results given the current corporate sustainability structures traversing HR and sustainability departments. Therefore, future work to validate this tool would be highly valuable to the advancement of corporate sustainability practices.

Although outlined as a limitation of the study, the virtual nature of this training provides a unique perspective of the positive impact of practitioners’ engagement with biomimicry and the natural world in a virtual manner. This provided a strong foundation for future research to explore the positive psychological potential of biomimicry exposure through a physical immersion in the natural world. Therefore, future research conducting a similar training experience in person with R&D employees with a specific focus on immersion in natural settings would be beneficial to better explore the potential psychological benefits of biomimicry training. It should also be noted that correlations between self-report measures and actual behavior can be quite modest. Therefore, future research measuring specific behavior changes would be advised. Further work is also warranted to explore the generalizability of the results found. For example, inferences drawn from interventions such as the biomimicry training offered to participants in this study would be strongest from a complete randomized design for assigning participants, but strategies to accomplish this in a corporate setting will continue to be challenging. This study also made several unique theoretical connections to a diversity of other domains such as marketing and human resources. Future work across these domains and the intersection between such and biomimicry would be a significant contribution to advance the field of biomimicry adoption and implementation within a corporate context.

## 6. Conclusions

In conclusion, the purpose of this study was to explore to the potential of biomimicry as job-specific training to intrinsically motivate R&D employees to reconnect with nature, promoting creative thinking and engagement in sustainability. Although not demonstrated in this study, this study outlined the theoretical basis for biomimicry, as a job-specific sustainability training opportunity, to increase employee’s connectedness with nature, fostering pro-environmental attitudes and behaviors [18,19,20,71] that could potentially create a more eco-centric-values-based corporate culture. Such a corporate culture is not only critical to the success of corporate sustainability goals but is also critical to attract and retain talent, especially among the incoming eco-conscious generation. However, the study results did show that biomimicry, as a job-specific professional development training opportunity, is intrinsically motivating to R&D employees and has the potential to create positive change across a diversity of departments within a corporate environment beyond its traditional innovation value. Biomimicry can be valued as a design approach to enhance sustainable innovation but also as a sustainable marketing campaign to increase employee engagement in sustainability, strengthening retention and recruitment, advancing corporate sustainability goals, and, as a result, the company’s bottom line [67].

This study also provides an adaptable procedural template and justification for organizations to experiment with biomimicry, illustrating its potential to create positive change across several business units.

## Figures and Tables

**Table 1:** Study design. This was repeated for each SBA, one per month from November to February. The pre-survey was identical for both treatment groups focused on “sustainable engagement opportunities”. The post-survey and 4-week survey for the no-training group remained unchanged. The post-survey and 4-week survey for the training group contained the same survey questions with slightly adapted terminology to focus on the effect of the “biomimicry training” (Appendix A.1).

Treatment	Day 1	Day 8	Day 10	4 Week
No Training	Pre-survey		Post-survey	4-Week Survey
Training	Pre-survey	*Biomimicry Training Workshop 1*	*Biomimicry Training Workshop 2* Post-survey	4-Week Survey

**Table 2:** Study participant demographics.

		No Training	Training
Participants (N)	Total	20	30
Gender:	Female	12	18
	Male	8	12
Age groups	18–29	45%	17%
	30–49	50%	56%
	50–64	5%	27%
Level of education	College graduate	55%	27%
	Graduate work	10%	10%
	Graduate gegree	35%	63%

**Table 3:** Repeated measures ANOVA results for the Intrinsic Motivation Inventory.

Source	DF	F Ratio	*p*	Effect Size (η^2^)
Treatment	1	3.859	0.0552	
Time	2	8.677	0.0004 *	**0.163**
Treatment x Time	2	8.004	0.0006 *	**0.152**

* Statistically significant result.

**Table 4:** Repeated measures ANOVA results for the Intrinsic Motivation Inventory subscale interest and enjoyment.

Source	DF	F Ratio	*p*	Effect Size (η^2^)
Treatment	1	5.220	0.0267 *	**0.097**
Time	2	9.993	0.0001 *	**0.183**
Treatment xTime	2	6.525	0.0023 *	**0.127**

* Statistically significant result.

**Table 5:** Repeated measures ANOVA results for the Intrinsic Motivation Inventory subscale value and usefulness.

Source	DF	F Ratio	*p*	Effect Size (η^2^)
Treatment	1	2.178	0.1465	
Time	2	4.830	0.0102 *	**0.098**
Treatment x Time	2	6.874	0.0017 *	**0.134**

* Statistically significant result.

**Table 6:** Alternative use test results of the mixed-model repeated measures ANOVA.

Source	DF	F Ratio	*p*	Effect Size
Treatment	1	1.853	0.1800	
Time	2	414.633	<0.0001 *	**0.254**
Treatment x Time	2	0.949	0.3911	

* Statistically significant result.

**Table 7:** Means, standard deviations, and Pearson correlation matrix.

Measures	M	SD	1	2	3	4	5	6
1. Gender	1.60	0.49						
2. Age	2.90	0.68	−0.24					
3. Alternative Use Test	2.48	0.33	0.18	−0.16	(0.39)			
4. Connectedness to Nature	3.48	0.57	0.09	0.25	−0.01	(0.12)		
5. Intrinsic Motivation Inventory	3.87	0.65	0.16	−0.23	−0.01	0.30 *	(0.21)	
6. Sustainability Engagement Index	3.77	0.71	0.12	−0.09	0.04	0.32 *	0.48 *	(0.13)

* *p* < 0.001.

## Data Availability

Restrictions apply to the availability of these data. Data was obtained from within a corporate entity and are available from the lead author SJM with the permission of the corporate entity.

## Appendix A

### Appendix A.1. **Participant Survey.** All Study Instruments Embedded

**Demographics (Only Asked in Pre-Survey for Both Treatment Groups)**	
Gender	Female Male
Age	17 and Under 18–29 30–49 50–64 65+
Highest Level of Education	College grad Some graduate work Graduate degree
Degree Earned	Open Comment
**All remaining questions were asked across all three time points Pre-, Post-, and 4-Week surveys for both treatment groups.**	
Subject Code: Please create a unique four digit code which will be used as your identifier to cross reference your survey responses.	X X X X
**Alternative Use Test**	
To start we are going to test your creative brain power through a little game. For this task, you should write down all the original and creative uses that you can think of for a common item, that you’ll be given. Certainly, there are common, unoriginal ways to use this item; but for this task, write down all the unusual, creative, and uncommon uses you can think of. You’ll have two minutes, and we’ll complete two tasks.	Stimulus items: Pre-Survey Images: a pen and a ping-pong ball. Post-Survey Images: a towel and bottle. 4-week Survey images: a brick and a paperclip.
**Connectedness to Nature Scale**	**Strongly disagree/** **Disagree/Neutral/Agree/Strongly Agree**
I often feel a sense of oneness with the natural world around me	1 2 3 4 5
I think of the natural world as a community to which I belong	1 2 3 4 5
I recognize and appreciate the intelligence of other living organisms	1 2 3 4 5
I often feel disconnected from nature (reverse scored)	1 2 3 4 5
When I think of my life, I imagine myself to be part of a larger cyclical process of living	1 2 3 4 5
I often feel a kinship with animals and plants	1 2 3 4 5
I Feel as though I belong to the Earth as equally as it belongs to me	1 2 3 4 5
I have a deep understanding of how my actions affect the natural world	1 2 3 4 5
I often feel part of the web of life	1 2 3 4 5
I feel that all inhabitants of Earth, human and nonhuman, share a common ‘life force’	1 2 3 4 5
Like a tree can be a part of a forest, I feel embedded within the broader natural world.	1 2 3 4 5
When I think of my place on Earth, I consider myself to be a top member of a hierarchy that exists in nature (reverse scored).	1 2 3 4 5
I often feel like I am only a small part of the natural world around me and that I am no more important than the grass on the ground or the birds in the trees	1 2 3 4 5
My personal welfare is independent of the welfare of the natural world	1 2 3 4 5
**Intrinsic Motivation Inventory** **No-training group terminology focused on *sustainability engagement opportunities* across all three time points.** **Training group terminology focused on *sustainability engagement opportunities* for the pre-survey and adapted to focus on *biomimicry training* for the post- and 4-week survey.**	
I very much enjoy the *sustainability engagement opportunities/Biomimicry training* available at my company	1 2 3 4 5
I believe these opportunities are of some value to me	1 2 3 4 5
These opportunities do not hold my attention at all (reverse scored).	1 2 3 4 5
I think current *sustainability engagement opportunities/Biomimicry training* are useful for design innovation	1 2 3 4 5
I would describe the *sustainability engagement opportunities/Biomimicry training* available at my company as very interesting	1 2 3 4 5
I think these opportunities are important to be involved in because they can promote environmental sustainability	1 2 3 4 5
Throughout these experiences I have thought about how much I enjoy them	1 2 3 4 5
Being involved in these *sustainability engagement opportunities/Biomimicry training* could help me be a better systems thinker	1 2 3 4 5
The *sustainability engagement opportunities/Biomimicry training* are fun to get involved in	1 2 3 4 5
I would be willing to get involved in *sustainability engagement opportunities/Biomimicry training* again because it has some value to me.	1 2 3 4 5
**Sustainability Engagement Index** **No-training group terminology focused on *sustainability engagement opportunities* across all three time points.** **Training group terminology focused on *sustainability engagement opportunities* for the pre-survey and adapted to focus on *biomimicry training* for the post- and 4-week survey.**	
*Sustainability engagement opportunities / Biomimicry training* available at my company demonstrate practices that can be incorporated into my daily job activities	1 2 3 4 5 (Open comment to elaborate answer)
Sustainability is critical to the future success of my company	1 2 3 4 5 (Open comment to elaborate answer)
The *sustainability engagement opportunities/Biomimicry training* available at work enhances my job satisfaction and overall feelings about the company	1 2 3 4 5 (Open comment to elaborate answer)
In my opinion, my company’s *sustainability engagement opportunities/Biomimicry training* enhance our brand in the community	1 2 3 4 5 (Open comment to elaborate answer)
The *sustainability engagement opportunities/Biomimicry training* available at my company demonstrate practices that can be incorporated into my personal life	1 2 3 4 5 (Open comment to elaborate answer)

### Appendix A.2. Alternative Use Test: Instructions for Judging Creativity of Answers

Creativity can be viewed as having three facets. Creative responses will generally be high on all three, although being low on one of them does not disqualify a response from getting a high rating. We will use a 1 to 5 scale: not at all creative (1) to highly creative (5).

1. Uncommon

Creative ideas are uncommon: they will occur infrequently in our sample. Any response that is given by a lot of people is common, by definition. Unique responses will tend to be creative responses, although a response given only once need not be judged as creative. For example, a random or inappropriate response would be uncommon but not creative.

2. Remote

Creative ideas are remotely linked to everyday objects and ideas. For example, creative uses for a brick are―far from common, everyday, normal uses for a brick, and creative instances of things that are round are―far from common round objects. Responses that stray from obvious ideas will tend to be creative, whereas responses close to obvious ideas will tend to be uncreative.

3. Clever

Creative ideas are often clever: they strike people as insightful, ironic, humorous, fitting, or smart. Responses that are clever will tend to be creative responses. Keep in mind that cleverness can compensate for the other facets. For example, a common use cleverly expressed could receive a high score.

## References

[1] National Environmental Education Foundation Winning in the Marketplace and the Workplace. https://www.neefusa.org/resource/winning-marketplace-and-workplace.

[2] Robertson J.L., Carleton E. (2018). Uncovering How and When Environmental Leadership Affects Employees’ Voluntary Pro-environmental Behavior. J. Leadersh. Organ. Stud..

[3] Turning Point: Corporate Progress on the Ceres Roadmap for Sustainability. https://www.ceres.org/resources/roadmap-for-sustainability.

[4] Yelon S., Sheppard L., Sleight D., Ford J.K. (2004). Intention to transfer: How do autonomous professionals become motivated to use new ideas?. Perform. Improv. Q..

[5] Boiral O., Paillé P., Raineri N., Robertson J.L., Barling J. (2015). The Nature of Employees’ Pro-Environmental Behaviors. The Psychology of Green Organizations.

[6] Unsworth K.L., Dmitrieva A., Adriasola E. (2013). Changing behaviour: Increasing the effectiveness of workplace interventions in creating pro-environmental behaviour change: GOALS AND PRO-ENVIRONMENTAL INTERVENTIONS. J. Organ. Behav..

[7] Deci E.L. (1971). Effects of externally mediated rewards on intrinsic motivation. J. Personal. Soc. Psychol..

[8] Kinley N., Ben-Hur S. (2015). Changing Employee Behavior.

[9] Pink D.H. (2011). Drive: The Surprising Truth about What Motivates Us.

[10] Pink D. The puzzle of motivation. Proceedings of the TEDGlobal 2009.

[11] Deci E.L., Eghrari H., Patrick B.C., Leone D.R. (1994). Facilitating internalization: The self-determination theory perspective. J. Personal..

[12] Wahl D.C. (2006). Bionics vs. biomimicry: From control of nature to sustainable participation in nature. Design and Nature III: Comparing Design in Nature with Science and Engineering.

[13] Zelenika I., Moreau T., Lane O., Zhao J. (2018). Sustainability education in a botanical garden promotes environmental knowledge, attitudes and willingness to act. Environ. Educ. Res..

[14] Zelenski J.M., Dopko R.L., Capaldi C.A. (2015). Cooperation is in our nature: Nature exposure may promote cooperative and environmentally sustainable behavior. J. Environ. Psychol..

[15] Rees W.E. (2002). Globalization and Sustainability: Conflict or Convergence?. Bull. Sci. Technol. Soc..

[16] Walker B., Holling C.S., Carpenter S.R., Kinzig A. (2004). Resilience, Adaptability and Transformability in Social–ecological Systems. Ecol. Soc..

[17] Ives C.D., Giusti M., Fischer J., Abson D.J., Klaniecki K., Dorninger C., Laudan J., Barthel S., Abernethy P., Martín-López B. (2017). Human–nature connection: A multidisciplinary review. Curr. Opin. Environ. Sustain..

[18] Nisbet E.K., Zelenski J.M., Murphy S.A. (2009). The nature relatedness scale: Linking individuals’ connection with nature to environmental concern and behavior. Environ. Behav..

[19] Dunlap R.E., Van Liere K.D., Mertig A.G., Jones R.E. (2000). Measuring Endorsement of the New Ecological Paradigm: A Revised NEP Scale. J. Soc. Issues.

[20] Schultz P.W. (2002). Inclusion with nature: The psychology of human-nature relations. Psychology of Sustainable Development.

[21] Schultz P.W. (2011). Conservation Means Behavior: Conservation Means Behavior. Conserv. Biol..

[22] Berman M.G., Jonides J., Kaplan S. (2008). The Cognitive Benefits of Interacting With Nature. Psychol. Sci..

[23] Kaplan S. (2000). New Ways to Promote Proenvironmental Behavior: Human Nature and Environmentally Responsible Behavior. J. Soc. Issues.

[24] Atchley R.A., Strayer D.L., Atchley P. (2012). Creativity in the Wild: Improving Creative Reasoning through Immersion in Natural Settings. PLoS ONE.

[25] Stephen L. (2021). The Deloitte Global Millennial Survey: A Decade in Review.

[26] Baumeister D., Tocke R., Dwyer J., Ritter S., Benyus J.M. (2014). Biomimicry Resource Handbook: A Seed Bank of Best Practices.

[27] Benyus J.M. (1997). Biomimicry: Innovation Inspired by Nature.

[28] (2015). Biomimetics—Terminology, Concepts and Methodology.

[29] Goel A.K. Biologically Inspired Design: A New Paradigm for AI Research on Computational Sustainability?. Proceedings of the Workshops at the Twenty-Ninth AAAI Conference on Artificial Intelligence, Computational Sustainability.

[30] Helms M., Vattam S.S., Goel A.K. (2009). Biologically inspired design: Process and products. Des. Stud..

[31] Yen J., Weissburg M. (2007). Perspectives on biologically inspired design: Introduction to the collected contributions. Bioinspir. Biomim..

[32] Faludi J., Ali O., Srour O., Mecanna S., Kamareddine R., Chatty T. (2019). Preliminary Results Testing What Different Design Solutions Arise from Different Sustainable Design Methods.

[33] Ilieva L., Ursano I., Traista L., Hoffmann B., Dahy H. (2022). Biomimicry as a Sustainable Design Methodology—Introducing the ‘Biomimicry for Sustainability’Framework. Biomimetics.

[34] Chirazi J., Wanieck K., Fayemi P.-E., Zollfrank C., Jacobs S. (2019). What do we learn from good practices of biologically inspired design in innovation?. Appl. Sci..

[35] Metz P., Burek S., Hultgren T.R., Kogan S., Schwartz L. (2016). The Path to Sustainability-Driven Innovation: Environmental sustainability can be the foundation for increasing competitive advantage and the basis for effective innovation. Res.-Technol. Manag..

[36] Kennedy E.B., Marting T.A. (2016). Biomimicry: Streamlining the Front End of Innovation for Environmentally Sustainable Products. Res.-Technol. Manag..

[37] Ives C.D., Abson D.J., von Wehrden H., Dorninger C., Klaniecki K., Fischer J. (2018). Reconnecting with nature for sustainability. Sustain. Sci.

[38] Harman J. (2013). The Shark’s Paintbrush: Biomimicry and How Nature Is Inspiring Innovation.

[39] McDonough W., Braungart M. (2010). Cradle to Cradle: Remaking the Way We Make Things.

[40] Fayemi P.-E., Wanieck K., Zollfrank C., Maranzana N., Aoussat A. (2017). Biomimetics: Process, tools and practice. Bioinspir. Biomim..

[41] Wanieck K., Fayemi P.-E., Maranzana N., Zollfrank C., Jacobs S. (2017). Biomimetics and its tools. Bioinspir. Biomim. Nanobiomater..

[42] Jacobs S.R., Nichol E.C., Helms M.E. (2014). “Where Are We Now and Where Are We Going?” The BioM Innovation Database. J. Mech. Des..

[43] Kennedy E.B. (2017). Biomimicry: Design by Analogy to Biology. Res.-Technol. Manag..

[44] Kennedy E., Niewiarowski P. (2018). Biomimicry: Do Frames of Inquiry Support Search and Identification of Biological Models?. Designs.

[45] Nagel J.K.S., Goel A.K., McAdams D.A., Stone R.B. (2014). A Thesaurus for Bioinspired Engineering Design. Biologically Inspired Design.

[46] Dicks H. (2016). The philosophy of biomimicry. Philos. Technol..

[47] Dicks H. (2017). Environmental ethics and biomimetic ethics: Nature as object of ethics and nature as source of ethics. J. Agric. Environ. Ethics.

[48] Dicks H. (2017). A New Way of Valuing Nature: Articulating Biomimicry and Ecosystem Services. Environ. Ethics.

[49] Vincent J.F.V., Bogatyreva O.A., Bogatyrev N.R., Bowyer A., Pahl A.-K. (2006). Biomimetics: Its practice and theory. J. R. Soc. Interface.

[50] Guilford J., Chrtstensen P., Wilson R., Merrifield P. (1960). Alternate Uses, Form A.

[51] Mayer F.S., Frantz C.M. (2004). The connectedness to nature scale: A measure of individuals’ feeling in community with nature. J. Environ. Psychol..

[52] McAuley E., Duncan T., Tammen V.V. (1989). Psychometric properties of the Intrinsic Motivation Inventory in a competitive sport setting: A confirmatory factor analysis. Res. Q. Exerc. Sport.

[53] Intrinsic Motivation Inventory (IMI). selfdeterminationtheory.org.

[54] McAuley E., Wraith S., Duncan T.E. (1991). Self-Efficacy, Perceptions of Success, and Intrinsic Motivation for Exercise 1. J. Appl. Soc. Psychol..

[55] Restall B., Conrad E. (2015). A literature review of connectedness to nature and its potential for environmental management. J. Environ. Manag..

[56] Salazar G., Monroe M.C., Jordan C., Ardoin N.M., Beery T.H. (2021). Improving Assessments of Connection to Nature: A Participatory Approach. Front. Ecol. Evol..

[57] Mayer F.S., Frantz C.M., Bruehlman-Senecal E., Dolliver K. (2009). Why Is Nature Beneficial?: The Role of Connectedness to Nature. Environ. Behav..

[58] Tam K.-P. (2013). Concepts and measures related to connection to nature: Similarities and differences. J. Environ. Psychol..

[59] Silvia P., Winterstein B., Willse J., Barona C., Cram J., Hess K., Martinez J., Richard C. (2008). Assessing Creativity With Divergent Thinking Tasks: Exploring the Reliability and Validity of New Subjective Scoring Methods. Psychol. Aesthet. Creat. Arts.

[60] Silvia P.J., Nusbaum E.C., Beaty R.E. (2017). Old or New? Evaluating the Old/New Scoring Method for Divergent Thinking Tasks. J. Creat. Behav..

[61] Benedek M., Neubauer A.C. (2013). Revisiting Mednick’s Model on Creativity-Related Differences in Associative Hierarchies. Evidence for a Common Path to Uncommon Thought. J. Creat. Behav..

[62] Amabile T.M. (1982). Social psychology of creativity: A consensual assessment technique. J. Personal. Soc. Psychol..

[63] Goldstein I.L., Ford J.K. (2002). Training in Organisations.

[64] Grossman R., Salas E. (2011). The transfer of training: What really matters: The transfer of training. Int. J. Train. Dev..

[65] Ryan R.M., Deci E.L. (2000). Intrinsic and Extrinsic Motivations: Classic Definitions and New Directions. Contemp. Educ. Psychol..

[66] Engaging Employees to Create a Sustainable Business (SSIR). https://ssir.org/articles/entry/engaging_employees_to_create_a_sustainable_business.

[67] Rudick T. New Business Imperative. White Paper. *EarthShare*. https://www.earthshare.org/earthshare-white-paper-the-new-business-imperative/n.

[68] Peterson M., Minton E.A., Liu R.L., Bartholomew D.E. (2021). Sustainable Marketing and Consumer Support for Sustainable Businsses. Sustain. Prod. Consum..

[69] Hennessey B.A., Amabile T.M. (2010). Creativity. Annu. Rev. Psychol..

[70] Wang X., Geng L., Zhou K., Ye L., Ma Y., Zhang S. (2016). Mindful learning can promote connectedness to nature: Implicit and explicit evidence. Conscious. Cogn..

[71] Nisbet E.K., Zelenski J.M., Murphy S.A. (2011). Happiness is in our nature: Exploring nature relatedness as a contributor to subjective well-being. J. Happiness Stud..

